# The C-Terminal Amino Acid of the MHC-I Heavy Chain Is Critical for Binding to Derlin-1 in Human Cytomegalovirus US11-Induced MHC-I Degradation

**DOI:** 10.1371/journal.pone.0072356

**Published:** 2013-08-12

**Authors:** Sunglim Cho, Bo Young Kim, Kwangseog Ahn, Youngsoo Jun

**Affiliations:** 1 Cell Dynamics Research Center and School of Life Sciences, Gwangju Institute of Science and Technology, Gwangju, Korea; 2 National Creative Research Initiatives Center for Antigen Presentation, Department of Biological Sciences, Seoul National University, Seoul, Korea; Oxford University, United Kingdom

## Abstract

Derlin-1 plays a critical role in endoplasmic reticulum-associated protein degradation (ERAD) of a particular subset of proteins. Although it is generally accepted that Derlin-1 mediates the export of ERAD substrates from the ER to the cytosol, little is known about how Derlin-1 interacts with these substrates. Human cytomegalovirus (HCMV) US11 exploits Derlin-1-dependent ERAD to degrade major histocompatibility complex class I (MHC-I) molecules and evade immune surveillance. US11 requires the cytosolic tail of the MHC-I heavy chain to divert MHC-I molecules into the ERAD pathway for degradation; however, the underlying mechanisms remain unknown. Here, we show that the cytosolic tail of the MHC-I heavy chain, although not required for interaction with US11, is required for tight binding to Derlin-1 and thus for US11-induced dislocation of the MHC-I heavy chain to the cytosol for proteasomal degradation. Surprisingly, deletion of a single C-terminal amino acid from the cytosolic tail disrupted the interaction between MHC-I molecules and Derlin-1, rendering mutant MHC-I molecules resistant to US11-induced degradation. Consistently, deleting the C-terminal cytosolic region of Derlin-1 prevented it from binding to MHC-I molecules. Taken together, these results suggest that the cytosolic region of Derlin-1 is involved in ERAD substrate binding and that this interaction is critical for the Derlin-1-mediated dislocation of the MHC-I heavy chain to the cytosol during US11-induced MHC-I degradation.

## Introduction

Nearly half of all human genes encode transmembrane or secreted proteins, most of which are co-translationally imported into the ER where they are folded into their native conformation. The ER operates a protein quality control system, which ensures that only correctly folded proteins are allowed to exit the ER for transport to their final destination within the cell or extracellular space [1–3]. By contrast, terminally misfolded and/or damaged proteins, which can be toxic to the cell, are rapidly exported to the cytosol and degraded by the proteasome via an evolutionarily conserved process, known as endoplasmic reticulum-associated protein degradation (ERAD) [4–6]. ERAD begins with recognition of ERAD substrates (i.e., proteins to be degraded via this process) present in the ER lumen or embedded in the ER membrane. These proteins are then directed to an as-yet poorly-defined protein complex, which is responsible for their retro-translocation or dislocation [6]. During dislocation, the ERAD substrates are exported to the cytosol where they are degraded by the ubiquitin-proteasome pathway.

Although degradation of ERAD substrates by the ubiquitin-proteasome pathway is relatively well characterized [7–9], little is known about how ERAD substrates are recognized and directed to the ERAD machinery, or about how the dislocation of ERAD substrates to the cytosol is executed and regulated. Since dislocation requires the transport of ERAD substrates across the ER membrane, it is assumed that a protein-conducting channel, or dislocon, may be involved in the process. This channel, however, is enigmatic [6]. Although there is no direct evidence for a channel, several lines of evidence suggest that the ER membrane protein, Derlin-1, may act as a protein-conducting channel for the ERAD of a subset of proteins: (i) Derlin-1 is essential for the dislocation of a subset of proteins during ERAD [10–12]; (ii) it is a transmembrane protein comprising transmembrane domains (TMDs), and can self-associate to yield multimeric complexes or it can associate with other ER membrane proteins [13,14]; and (iii) it binds its ERAD substrates both before and after dislocation [10,15,16]. However, a recent study by Greenblatt et al. [15] suggests that, rather than acting as a channel, Derlin-1 is an inactive member of the rhomboid family of intramembrane proteases. Based on homology modeling analyses of Derlin-1 and the bacterial rhomboid protease, GlpG (the only member on this protein family for which a high resolution structure has been identified), Greenblatt et al. argued that Derlin-1 is unlikely to form a channel because its predicted structure does not suggest an obvious pathway through the membrane. Consistent with the notion that Derlin-1 may be both a member of the rhomboid protease family and involved in ERAD, some rhomboid proteases were reported to regulate the dislocation process during ERAD. For example, the inactive rhomboids, iRhom-1 and iRhom-2, stimulate the ERAD of EGFR ligands in *Drosophila* [17]. Also, RHBDL4, an ubiquitin-dependent intramembrane rhomboid protease, mediates the degradation of various membrane proteins via ERAD [18].

The human cytomegalovirus (HCMV) gene products, US2 and US11, exploit the ERAD pathway to degrade major histocompatibility complex class I (MHC-I) molecules and avoid immune surveillance [19]. US2 and US11 physically bind to newly-synthesized MHC-I heavy chains and induce their dislocation to the cytosol for degradation by the proteasome [20,21]. Although both of these viral proteins target the same proteins for cytosolic degradation, they employ mutually exclusive pathways. US2 requires the non-proteolytic function of signal peptide protease (SPP) to dislocate MHC-I molecules [22] and does not require Derlin-1 [10]. By contrast, US11-induced degradation of MHC-I molecules requires Derlin1 but not SPP [10,22]. The ER luminal domain of US11 interacts with the luminal domain of the MHC-I heavy chain, whereas the TMD of US11 binds to Derlin-1. Thus, the major function of US11 is likely to be the delivery of MHC-I molecules to Derlin-1 [10,16], which then induces their dislocation to the cytosol for proteasomal degradation. In addition, US11 activates the unfold protein response, which switches on the expression of genes that facilitate the ERAD pathway [23].

Derlin-1 forms part of multi-protein complex that mediates the dislocation, ubiquitination, and extraction of ERAD substrates from the ER membrane [13,14]. Thus, once captured by Derlin-1, MHC-I molecules are rapidly dislocated, ubiquitinated by an as-yet unidentified E3 ligase [24] and, finally, extracted from the ER membrane and released into the cytosol by the cytosolic AAA ATPase p97, which provides the energy required for the process [15,25]. We recently found that replacing the TMD of MHC-I molecules with that of US11 forced them to interact with Derlin-1, whereupon they were rapidly dislocated to the cytosol and subsequently degraded by the proteasome [16]. This finding is consistent with a central role of Derlin-1 in this type of ERAD pathway and suggests that the interaction between Derlin-1 and its ERAD substrates is the key step that initiates protein dislocation and the subsequent steps of the ERAD pathway; thus Derlin-1 plays a key role in determining the final fate of its ERAD substrates. Surprisingly, despite its extreme importance, little is known about how Derlin-1 interacts with its ERAD substrates prior to or during dislocation. Using this model, here, we investigated the mechanism by which Derlin-1 interacts with its substrates. The results show that the cytosolic interaction between Derlin-1 and MHC-I molecules is critical for US11-induced dislocation of the MHC-I heavy chain to the cytosol for proteasomal degradation.

## Materials and Methods

### Cell culture and transfection

U373MG and HeLa cells were cultured in DMEM (Hyclone) supplemented with 7% fetal bovine serum (Hyclone), 2 mM Glutamax-I (Invitrogen), 100 U/ml penicillin (Invitrogen), and 100 μg/ml streptomycin (Invitrogen). Cells were transfected with Lipofectamine-2000 (Invitrogen). All assays were performed 36–48 hr after transfection. U373MG cells stably expressing HCMV US2 (U373MG-US2) or US11 (U373MG-US11) have been previously described [26].

### Plasmids

The plasmids pcDNA3.1-HLA-A2, pcDNA3.1-HLA-A2-ΔCT, pcDNA3.1-HLA-G, pcDNA3.1-GGA, pcDNA3.1-GGB, pcDNA3.1-GGC, pcDNA3.1-AUA, pcDNA3.1-GUA, pUHD10.1-US11, and pUHD10.1-US11-Q192L have been previously described [16,27] and were used as templates for generating the chimeras. The AUA-ΔCT, GUA-ΔCT, AUA-ΔV, and GUA-ΔV constructs were generated by PCR with primers carrying the respective mutations, and the resulting PCR products were inserted into pcDNA3.1 (Invitrogen) or pcDNA3.1-puro [27]. Derlin-1 was cloned from HeLa cell mRNA by RT-PCR and inserted into the plasmid, pEGFP-N3 (Clontech), to incorporate a C-terminal green fluorescent protein (GFP) tag. Derlin-1 deletion mutants were generated by PCR using specific primers and then sub-cloned into pEGFP-N3.

### Antibodies

Monoclonal antibody (mAb) BB7.2 and mAb HC10 were purified from the supernatant of mouse hybridoma cells. Monoclonal antibodies 4H84 (Santa Cruz) and G233 (Abcam) recognize denatured HLA-G heavy chains and assembled HLA-G/β_2_m, respectively. Anti-p97, anti-US11, and anti-Derlin-1 antibodies were previously described [16]. Anti-HSP90 and anti-GAPDH were purchased from Santa Cruz Biotechnology and AbFrontier, respectively. Monoclonal antibody HCA2, which reacts preferentially with HLA-A locus heavy chains, was a generous gift from Dr. Hidde Ploegh (Whitehead Institute, Cambridge, MA).

### Pulse-chase and immunoprecipitation

Pulse-chase and immunoprecipitation were performed as previously described [27]. Briefly, for pulse-chase experiments, cells were starved for 1 hr in methionine/cysteine-free DMEM (Invitrogen) and then labeled with ^35^S-methionine/cysteine (PerkinElmer) for 15 min. Unincorporated ^35^S-methionine/cysteine was removed by washing the cells three times with warm PBS. The cells were then transferred to normal medium for the chase. After incubating for indicated times, the cells were harvested and lysed with 1% NP-40 (Calbiochem) in PBS supplemented with 1 mM PMSF (Sigma) and 10 μM leupeptin (Calbiochem) for 30 min at 4° C. Post-centrifugation supernatants were pre-cleared by incubating them with protein G sepharose (PGS, Amersham) for 1 hr at 4° C. The pre-cleared supernatants were then incubated with 1 μg of indicated antibodies overnight at 4° C. After the addition of PGS, the supernatants were incubated for a further 1 hr and the material bound to PGS was precipitated, washed three times with 1% NP-40 in PBS, and eluted by boiling in SDS-sample buffer for 10 min. The samples were then separated in 10% SDS-PAGE gels and analyzed by autoradiography with a phosphorimager (FLA7000, Fujifilm). For endoglycosidase H (EndoH) analysis, the material bound to PGS was eluted by boiling in EndoH buffer (50 mM sodium acetate, pH 5.6, 0.3% SDS, and 150 mM β-mercaptoethanol) for 10 min. Each eluate was divided into two aliquots, which were incubated overnight at 37° C in the presence or absence of EndoH (5 mU, Roche).

### Sequential co-immunoprecipitation

Transfected cells were starved in methionine/cysteine-free DMEM for 1 hr and then labeled with ^35^S-methionine/cysteine for 1 hr. The cells were then lysed with 1% digitonin buffer (1% digitonin in 25 mM HEPES [pH 7.4], 150 mM NaCl, 1 mM PMSF, and 10 μM leupeptin). Post-centrifugation supernatants were pre-cleared with PGS for 1 hr at 4 °C. The pre-cleared supernatants were incubated with the indicated antibodies overnight at 4 °C. After adding PGS, the samples were incubated for a further 1 hr. The material bound to PGS was precipitated, washed twice with 0.1% digitonin buffer, and eluted in 100 μl of Re–IP buffer (1.5% SDS and 2 mM DTT in PBS). The eluates were diluted in 900 μl of 1% NP-40 in PBS and incubated with the indicated antibodies overnight at 4° C. PGS was then added, and the incubation was continued for 1 hr. The material bound to PGS was washed three times with 1% NP-40 in PBS and eluted by boiling in SDS-sample buffer for 10 min. Samples were separated in SDS-PAGE gels and analyzed by autoradiography.

## Results

### The cytosolic tail of the MHC-I heavy chain is required for strong binding to Derlin-1

**Figure 1:**
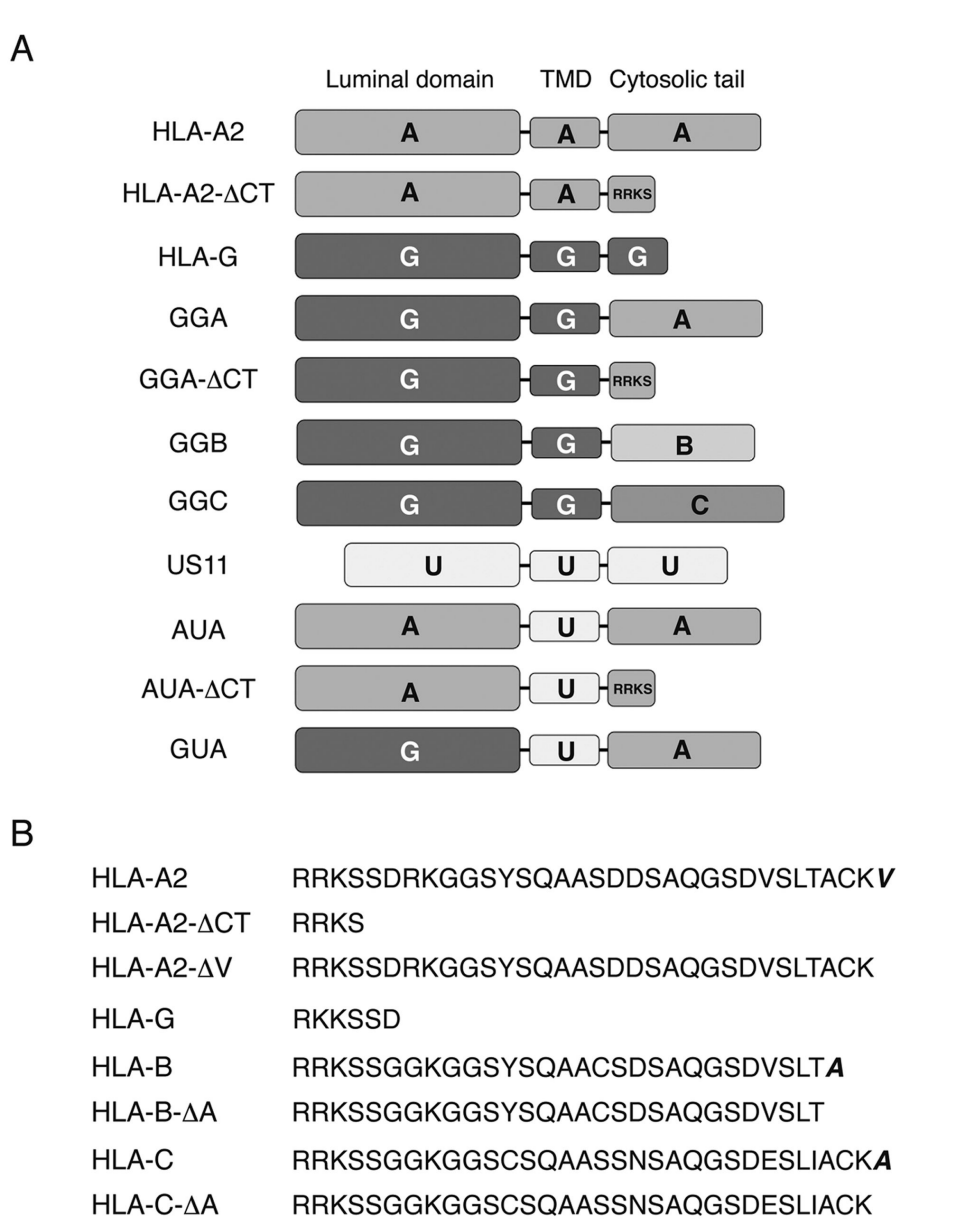
Protein constructs used in this study. (A) Schematic representation of the protein constructs used in this study. TMD, transmembrane domain (B) The amino acid sequence of the MHC-I heavy chain cytosolic tails examined in this study. The HLA alleles HLA-A0201, HLA-B1503, and HLA-Cw0602 were used for HLA-A2, HLA-B, and HLA-C, respectively.

**Figure 2:**
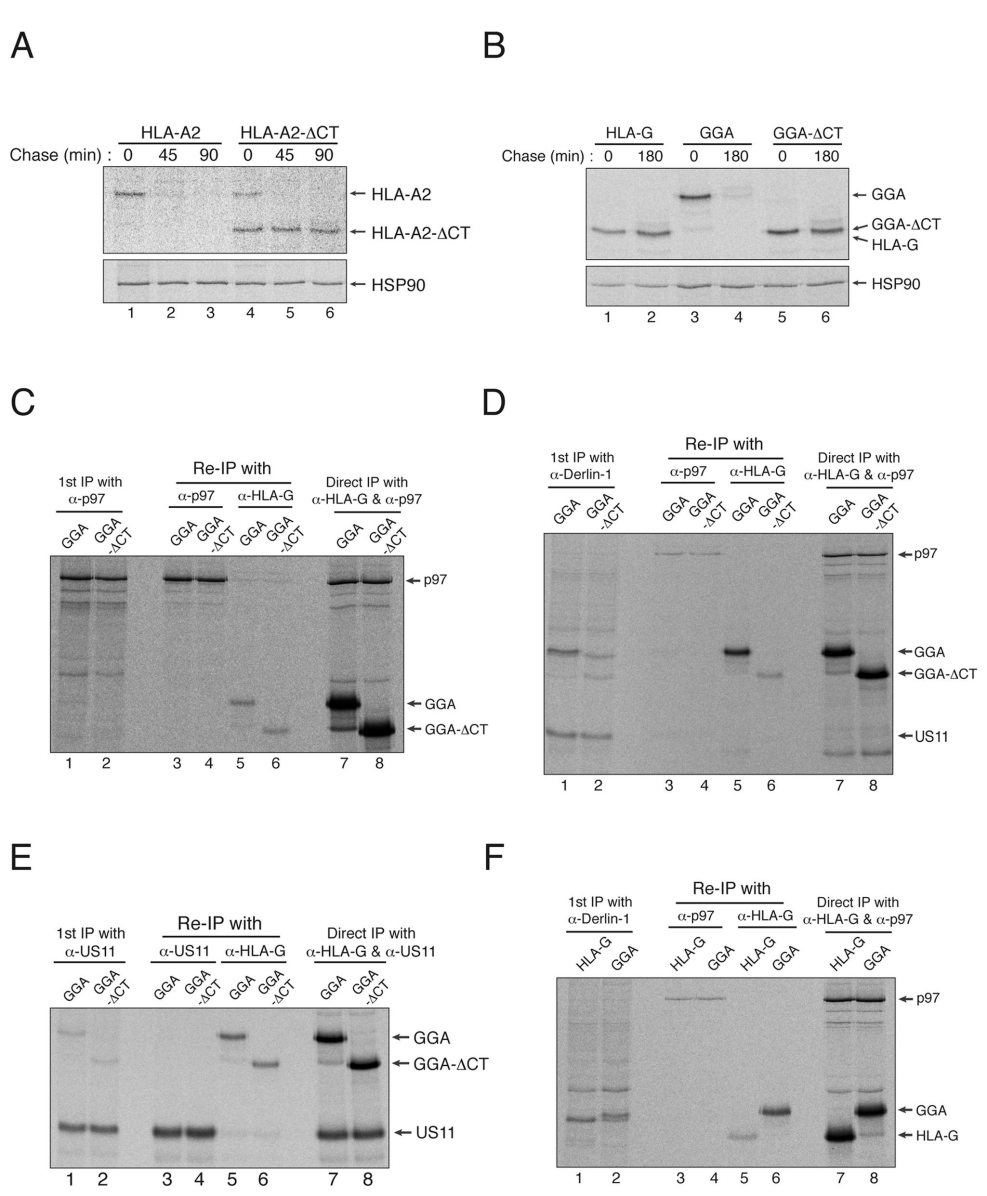
The cytosolic tail of an MHC-I molecule is required for strong binding to Derlin-1 during US11-induced ERAD. (A) Deletion of the cytosolic tail of HLA-A2 delays US11-induced degradation. U373MG-US11 cells were transfected with wild-type HLA-A2 or HLA-A2-ΔCT, metabolically labeled with ^35^S methionine/cysteine for 15 min, and chased for 0, 45, or 90 min. HLA-A2 or HLA-A2-ΔCT was recovered by immunoprecipitation with mAb BB7.2, separated by SDS-PAGE, and analyzed by autoradiography. Immunoprecipitation of HSP90 (lower panel) shows that the same amount of cell lysate was loaded into each lane of the gel. (B) A short cytosolic tail renders HLA-G resistant to US11-induced degradation. U373MG-US11 cells were transfected with wild-type HLA-G, GGA, or GGA-ΔCT, metabolically labeled for 15 min, and then chased for 0 or 180 min. HLA-G, GGA, or GGA-ΔCT was recovered by immunoprecipitation with mAb G233. (C) Deletion of the MHC-I heavy chain cytosolic tail has little effect on its interaction with p97. U373MG-US11 cells were transfected with GGA or GGA-ΔCT, metabolically labeled for 1 hr, lysed in 1% digitonin, and then subjected to immunoprecipitation with anti-p97 antibody. The precipitate was then boiled in SDS/DTT-containing buffer to disrupt all protein–protein interactions, diluted 10-fold in 1% NP-40, and then subjected to a second round of immunoprecipitation with the anti-p97 antibody or with mAb 4H84. (D) Deletion of the MHC-I cytosolic tail reduces its interaction with Derlin-1. U373MG-US11 cells were transfected with GGA or GGA-ΔCT, metabolically labeled for 1 hr, lysed in 1% digitonin, and subjected to immunoprecipitation with an anti-Derlin-1 antibody. The precipitate was then boiled in SDS/DTT-containing buffer, diluted 10-fold in 1% NP-40, and then subjected to a second round of immunoprecipitation with the anti-p97 antibody or with mAb 4H84. Re-immunoprecipitation of p97 indicates that the precipitation efficiency of Derlin-1 was likely to be comparable between samples. (E) Deletion of the MHC-I cytosolic tail does not affect its interaction with US11. The same digitonin-solubilized lysates described in (D) were subjected to immunoprecipitation with an anti-US11 antibody. The precipitate was then boiled in SDS/DTT-containing buffer, diluted 10-fold in 1% NP-40, and then subjected to a second round of immunoprecipitation with the anti-US11 antibody or with mAb 4H84. (F) HLA-G shows limited affinity for Derlin-1 because of its short cytosolic tail. All experiments were performed multiple times with similar results, and the data shown are representative of all results.

The cytosolic tail of the MHC-I heavy chain is required for HCMV US11-induced degradation [28,29]; however, the underlying mechanism remains unknown. To confirm previous observations, we first examined the two different human MHC-I molecules used in the previous studies: HLA-A2 and HLA-G [28–30]. To examine whether deleting the cytosolic tail of the HLA-A2 heavy chain affects US11-induced degradation, U373MG cells stably expressing HCMV US11 (U373MG-US11 cells) were transfected with wild-type HLA-A2 or with a tail-less mutant of HLA-A2 (HLA-A2-ΔCT) in which the entire cytosolic tail (apart from the four amino acids immediately proximal to the membrane) was deleted (Figure 1A). We then compared their half-lives in pulse-chase experiments (Figure 2A). While wild-type HLA-A2 was rapidly degraded over the chase time in US11-expressing cells, HLA-A2-ΔCT remained largely resistant to US11-mediated degradation (Figure 2A; compare lanes 3 and 6), which confirmed the results of previous studies. The difference in stability between the two proteins was even more evident in U373MG-US11 cells transfected with HLA-A2-ΔCT: transfected HLA-A2-ΔCT remained stable for 90 min whereas endogenously expressed HLA-A2 was rapidly degraded (Figure 2A; lanes 4–6, compare the upper and lower bands). HLA-G, a non-classical MHC-I molecule specifically expressed in the placenta, possesses an intrinsically short cytosolic tail, which confers resistance to US11-mediated degradation (Figure 2B; lanes 1–2). Consistent with the concept that an extended cytosolic tail is required for US11-mediated degradation, a hybrid HLA-G (GGA) molecule (in which the cytosolic tail was replaced with that of HLA-A2) was susceptible to US11-induced degradation (Figure 2B; lanes 3–4); However, deletion of the cytosolic tail rendered GGA resistant to degradation (Figure 2B; lanes 5–6). The slower degradation kinetics of GGA compared with those of HLA-A2 are consistent with the previous finding that the ER luminal domain of HLA-G influences its sensitivity to US11-induced degradation [29].

To identify the molecular mechanisms underlying the requirement of a cytosolic tail during the US11-induced degradation of MHC-I molecules, we first examined whether deleting the cytosolic tail affects molecular interactions between MHC-I molecules and essential components of the Derlin-1-dependent ERAD pathway, such as p97 and Derlin-1. Since p97 physically binds to MHC-I heavy chains and is essential for their dislocation [25], the cytosolic tail of the MHC-I heavy chain may serve as a point of contact for this cytosolic ATPase. To test this possibility, we examined whether deleting the cytosolic tail of GGA affected its association with p97. U373MG-US11 cells were transfected with either GGA or GGA-ΔCT, metabolically labeled for 1 hr, lysed in 1% digitonin, and then immunoprecipitated with an anti-p97 antibody (Figure 2C; lanes 1 and 2). The precipitate was then boiled in SDS/DTT-containing buffer to disrupt all protein–protein interactions and diluted 10-fold in 1% NP-40 before a second round of immunoprecipitation with the anti-p97 antibody (lanes 3 and 4) or with mAb 4H84 for denatured forms of HLA-G (lanes 5 and 6). The precipitated proteins were then separated in 10% SDS-PAGE gels and analyzed by autoradiography. The results showed that the interaction between p97 and GGA-ΔCT was similar to that between p97 and GGA (Figure 2C; compare lanes 5 and 6), indicating that the entire cytosolic tail may not be required for binding to p97 during US11-induced degradation of MHC-I molecules.

Next, we examined whether deleting the cytosolic tail of the MHC-I heavy chain affected its interaction with Derlin-1. To this end, we performed sequential co-immunoprecipitation experiments as described above. Because it has been reported that endogenous Derlin-1 is not readily detectable by metabolic labeling and autoradiography presumably due to its slow turnover rate [10,16], p97, a Derlin-1-binding protein during ERAD, was used to show that precipitation efficiency was comparable between samples (Figure 2D; lanes 3 and 4). Surprisingly, deleting the cytosolic tail markedly reduced the interaction between GGA and Derlin-1 (Figure 2D; compare lanes 5 and 6). Since it was thought that MHC-I molecules interact with Derlin-1 via US11 (US11 binds to the MHC-I heavy chain and Derlin-1 via its ER luminal domain and TMD, respectively) [10,31], the deletion of the MHC-I cytosolic tail would be expected to weaken the interaction between US11 and MHC-I molecules. Thus, we examined whether deleting the cytosolic tail of GGA would abrogate its interaction with US11. As shown in Figure 2E, deleting the cytosolic tail of GGA did not reduce the interaction between MHC-I and US11, suggesting that the cytosolic tail of the MHC-I heavy chain is critical for its interaction with Derlin-1. Consistent with this, HLA-G (which has an intrinsically short cytosolic tail) showed only marginal affinity for Derlin-1, whereas adding the HLA-A2 tail to HLA-G markedly increased its interaction with Derlin-1 (Figure 2F; compare lanes 5 and 6). Taken together, these results suggest that the cytosolic tail of the MHC-I heavy chain is required for US11-induced degradation because it enables MHC-I molecules to bind tightly to Derlin-1.

### The C-terminal amino acid of the MHC-I heavy chain is critical for binding to Derlin-1 and for subsequent degradation

**Figure 3:**
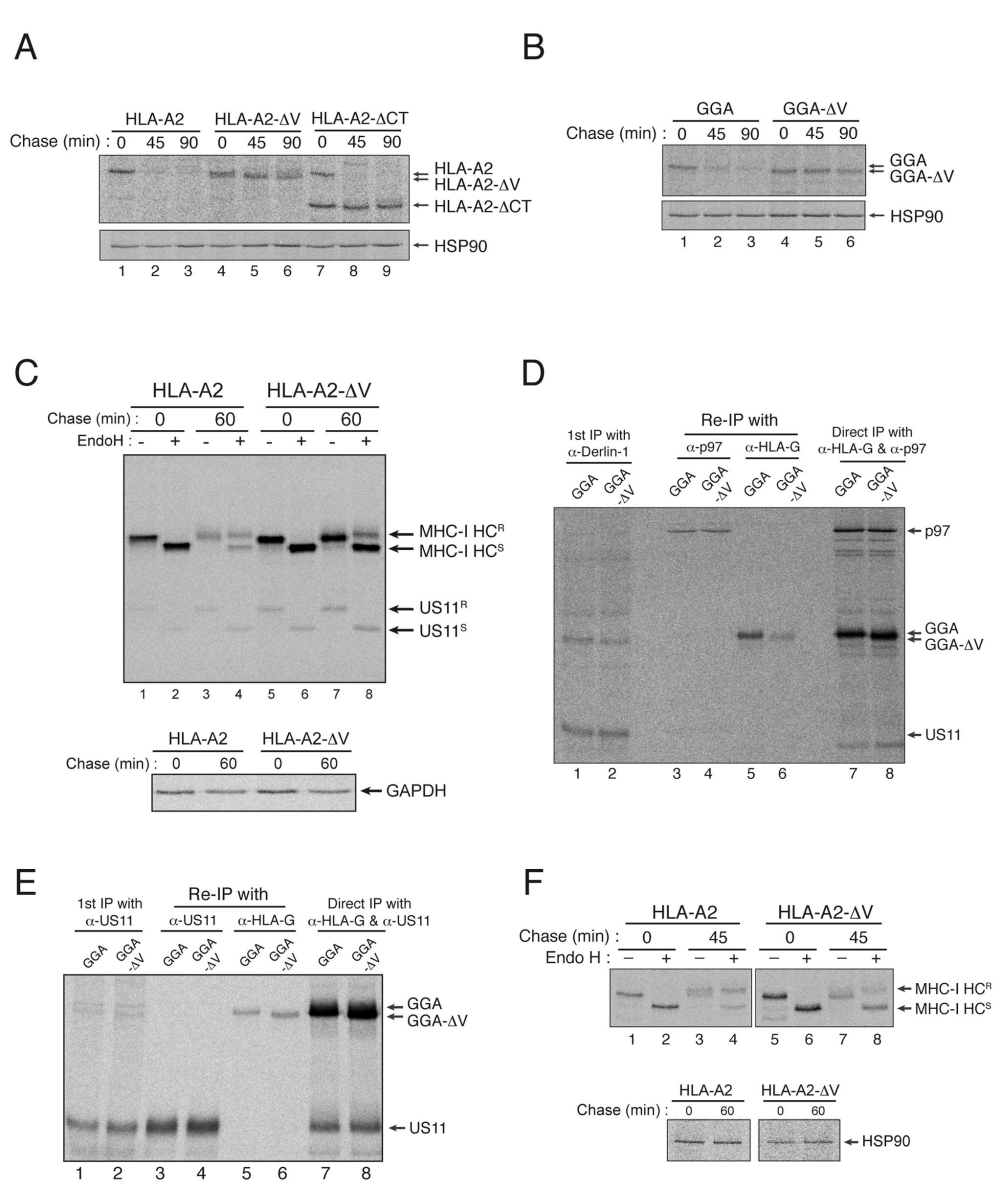
The C-terminal amino acid of the MHC-I heavy chain is critical for its interaction with Derlin-1 during US11-induced ERAD. (A) Deletion of a single C-terminal amino acid renders HLA-A2 resistant to US11-induced degradation. U373MG-US11 cells were transfected with wild-type HLA-A2, HLA-A2-ΔV or HLA-A2-ΔCT, metabolically labeled for 15 min, and then chased for 0, 45, or 90 min. (B) Deleting the C-terminal amino acid from the GGA construct blocks its degradation by US11. U373MG-US11 cells were transfected with GGA or GGA-ΔV, metabolically labeled for 15 min, and then chased for 0, 45, or 90 min. (C) The C-terminal valine of HLA-A2 is critical for HLA-A2 dislocation to the cytosol during US11-induced degradation. U373MG-US11 cells were transfected with either wild-type HLA-A2 or HLA-A2-ΔV, pulse-labeled for 15 min, and chased for 0 or 60 min. After lysis, HLA-A2 or HLA-A2-ΔV was recovered by immunoprecipitation with mAb BB7.2 and either left untreated or treated with EndoH (upper panel). Comparable levels of GAPDH show that the amount of the cell lysate used was equal between samples (lower panel). MHC-I HC^R^, EndoH-resistant MHC-I heavy chain; MHC-I HC^S^, EndoH-sensitive MHC-I heavy chain. (D) Deletion of the C-terminal valine residue disrupts the interaction between GGA and Derlin-1. U373MG-US11 cells were transfected with GGA or GGA-ΔV, metabolically labeled for 1 hr, lysed in 1% digitonin, and subjected to immunoprecipitation with the anti-p97 antibody. The precipitate was then boiled in SDS/DTT-containing buffer, diluted 10-fold in 1% NP-40, and then subjected to a second round of immunoprecipitation with the anti-p97 antibody or with mAb 4H84. (E) Deletion of the C-terminal valine residue of GGA does not affect its interaction with US11. The same digitonin-solubilized lysates described in (D) were subjected to immunoprecipitation with an anti-US11 antibody. The precipitate was then boiled in SDS/DTT-containing buffer, diluted 10-fold in 1% NP-40, and subjected to a second round of immunoprecipitation with the anti-US11 antibody or with mAb 4H84. (F) The C-terminal valine residue of HLA-A2 is not required for HCMV US2-induced degradation. U373MG-US2 cells were transfected with HLA-A2 or HLA-A2-ΔV, metabolically labeled for 15 min, and then chased for 0 or 45 min. HLA-A2 or HLA-A2-ΔV was recovered by immunoprecipitation with mAb BB7.2 and either left untreated or treated with EndoH (upper panel). Similar levels of HSP90 indicate that the amount of the cell lysate used was comparable between samples (lower panel). All experiments were performed multiple times with similar results, and the data shown are representative of all results.

To identify the critical region within the MHC-I cytosolic tail that is responsible for binding to Derlin-1 and subsequent degradation, we constructed a series of the C-terminal HLA-A2 deletion mutants and examined their susceptibility to US11-mediated degradation (data not shown). Strikingly, we found that deleting a single C-terminal amino acid from the HLA-A2 heavy chain (the valine residue in this case) affected its susceptibility to US11-induced degradation in a manner similar to that observed after deleting the entire cytosolic tail (Figure 3A). Indeed, the half-life of HLA-A2-ΔV was comparable with that of HLA-A2-ΔCT in US11-expressing cells (Figure 3A; compare lanes 4–6 and 7–9). Likewise, deleting the C-terminal valine residue rendered GGA resistant to US11-induced degradation (Figure 3B; compare lanes 1–3 and 4–6). These results are consistent with a previous report that the last two amino acids located at the C-terminus of the MHC-I heavy chain are important for US11-mediated degradation [29]. Unfolding or loss of β_2_-microglobulin, instead of degradation, may also reduce the amount of HLA-A2 and HLA-A2-ΔV molecules recovered by immunoprecipitation with mAb BB7.2 that recognizes HLA-A2 in a conformation-dependent manner (Figure 3A). Thus, mAb HCA2, which reacts preferentially with HLA-A locus heavy chains, was employed to confirm the insensitivity of HLA-A2-ΔV to US11-mediated degradation (Figure S1A). How could the deletion of the C-terminal amino acid possibly confer MHC-I molecules with considerable resistance to US11-induced degradation? We first examined whether the C-terminal amino acid of the MHC-I heavy chain is crucial for its dislocation from the ER membrane to the cytosol during US11-induced degradation. Because the N-linked glycan moiety of the MHC-I heavy chain is removed by an N-glycanase in the cytosol after its dislocation, which would expose the ER luminal domain of the MHC-I heavy chain to the cytosol, we examined whether HLA-A2-ΔV heavy chain retained the N-linked glycan in the presence of US11 by performing EndoH analysis. As shown in Figure 3C, after a 60-min chase, HLA-A2-ΔV still retained the N-linked glycan moiety (compare lanes 7 and 8), indicating that deletion of the C-terminal valine prevented US11-induced dislocation of HLA-A2 to the cytosol. Thus, the C-terminal valine of HLA-A2 appears to be required for dislocation to the cytosol under conditions of US11-induced degradation. This finding raised the question of whether the C-terminal amino acid is also critical for the interaction between MHC-I molecules and Derlin-1, which mediates MHC-I dislocation to the cytosol in the presence of US11. To address this, we examined whether deleting the C-terminal amino acid of GGA affected its interaction with Derlin-1. We found that deleting the C-terminal valine residue of GGA markedly weakened its interaction with Derlin-1 (Figure 3D; compare lanes 5 and 6). By contrast, deleting the C-terminal valine residue from GGA did not affect its interaction with US11 (Figure 3E). The weakened interaction between GGA-ΔV and Derlin-1 correlated well with the observed resistance of GGA-ΔV to US11-induced degradation (Figure 3B); thus the C-terminal amino acid of an MHC-I heavy chain seems to be required for US11-induced degradation because it plays a key role in binding to Derlin-1. This requirement for a C-terminal valine residue is specific to Derlin-1-dependent degradation, as this residue is not required for degradation induced by another HCMV protein, US2 (Figure 3F), which functions independently of Derlin-1 but requires the MHC-I cytosolic tail to degrade MHC-I molecules [10,22,28]. Taken together, the C-terminal amino acid of the MHC-I heavy chain is critical for binding to Derlin-1 and thereby for the subsequent dislocation and degradation of MHC-I molecules during US11-induced ERAD.

**Figure 4:**
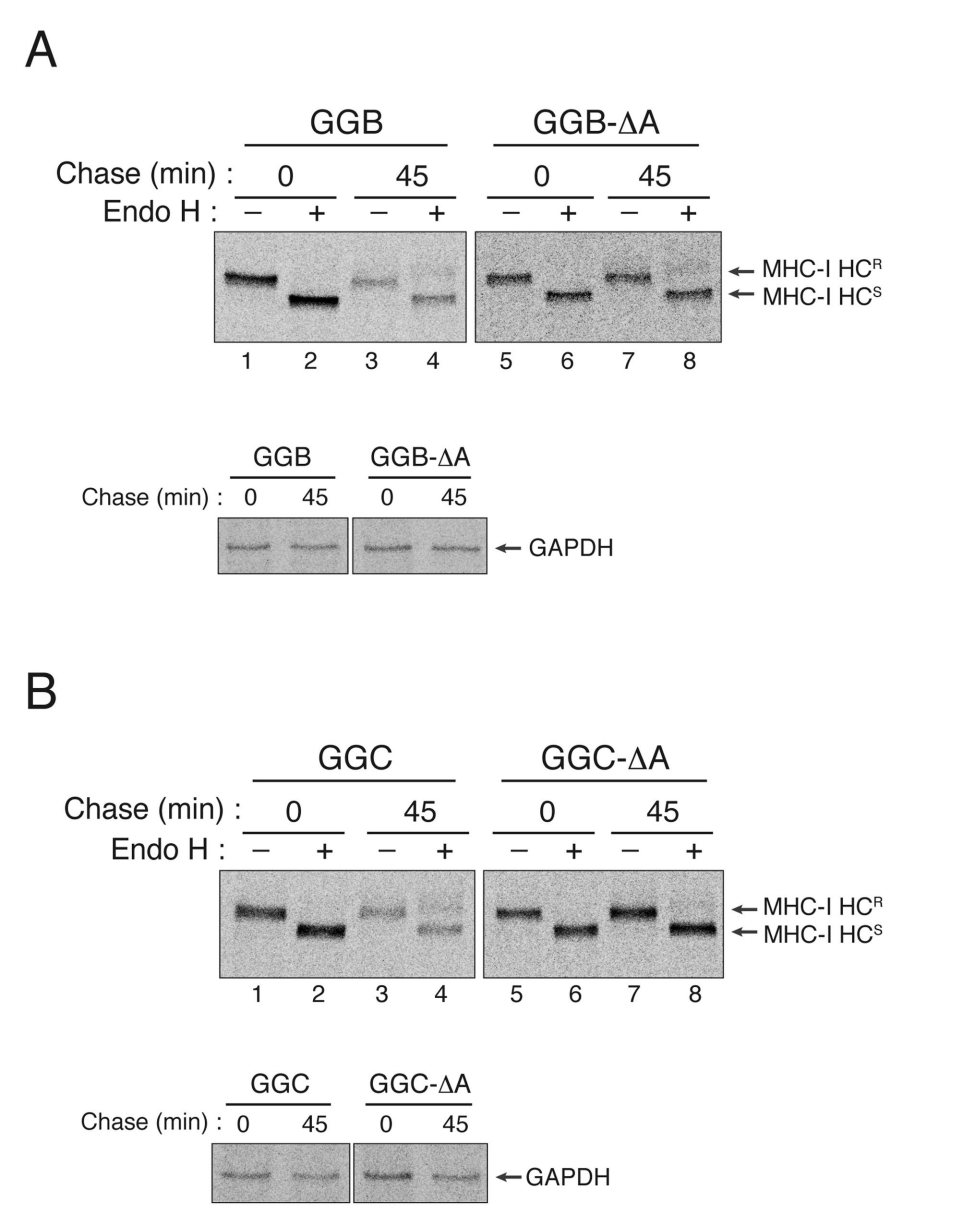
The C-terminal amino acids of HLA-B and HLA-C are critical for US11-induced degradation. (A) Deletion of the C-terminal alanine renders GGB resistant to US11-induced degradation. U373MG-US11 cells were transfected with GGB or GGB-ΔA, metabolically labeled for 15 min, and chased for 0 or 45 min. GGB or GGB-ΔA was recovered by immunoprecipitation with mAb G233 and either left untreated or treated with EndoH. GAPDH was used as loading control. (B) Deletion of the C-terminal alanine residue stabilizes GGC in US11-expressing cells. U373MG-US11 cells were transfected with GGC or GGC-ΔA, metabolically labeled for 15 min, and chased for 0 or 45 min. All experiments were performed multiple times with similar results, and the data shown are representative of all results.

Although MHC-I molecules are highly polymorphic, the C-terminal amino acid of the heavy chain is well conserved between different alleles: HLA-A alleles terminate in a valine residue whereas HLA-B and HLA-C alleles terminate in an alanine residue [27]. Because of this high degree of conservation, the C-terminal amino acid of MHC-I molecules is thought to be involved in allele-independent functions. Consistent with this idea, we recently reported that the C-terminal amino acid of the MHC-I heavy chain serves as a signal for ER export [27]. Thus, HCMV US11 may have evolved to target different MHC-I alleles by driving Derlin-1 to bind to the well-conserved C-terminal amino acid, regardless of the MHC-I alleles expressed by infected individuals. This strategy may enable HCMV to target various MHC-I alleles with a limited number of viral proteins. To examine whether the C-terminal alanine residue of the HLA-B and HLA-C heavy chains is also important for US11-induced degradation, we compared the half-lives of the GGB (in which the cytosolic tail of HLA-G was replaced with that of HLA-B1503) and GGC (in which the cytosolic tail of HLA-G was replaced with that of HLA-Cw0602) constructs (see Figure 1) with those of GGB-ΔA and GGC-ΔA, respectively, in the presence of US11 (Figure 4). Interestingly, deleting the C-terminal alanine residue markedly stabilized both GGB (Figure 4A; compare lanes 1–4 and lanes 5–8) and GGC (Figure 4B; compare lanes 1–4 and lanes 5–8) in US11-expressing cells. Thus, these results suggest that the well-conserved C-terminal amino acid (valine or alanine) of the human MHC-I heavy chain may be the target of Derlin-1 recognition during the US11-induced ERAD of MHC-I molecules.

### The cytosolic tail of the MHC-I heavy chain is not required for degradation when MHC-I molecules are forced to interact with Derlin-1

**Figure 5:**
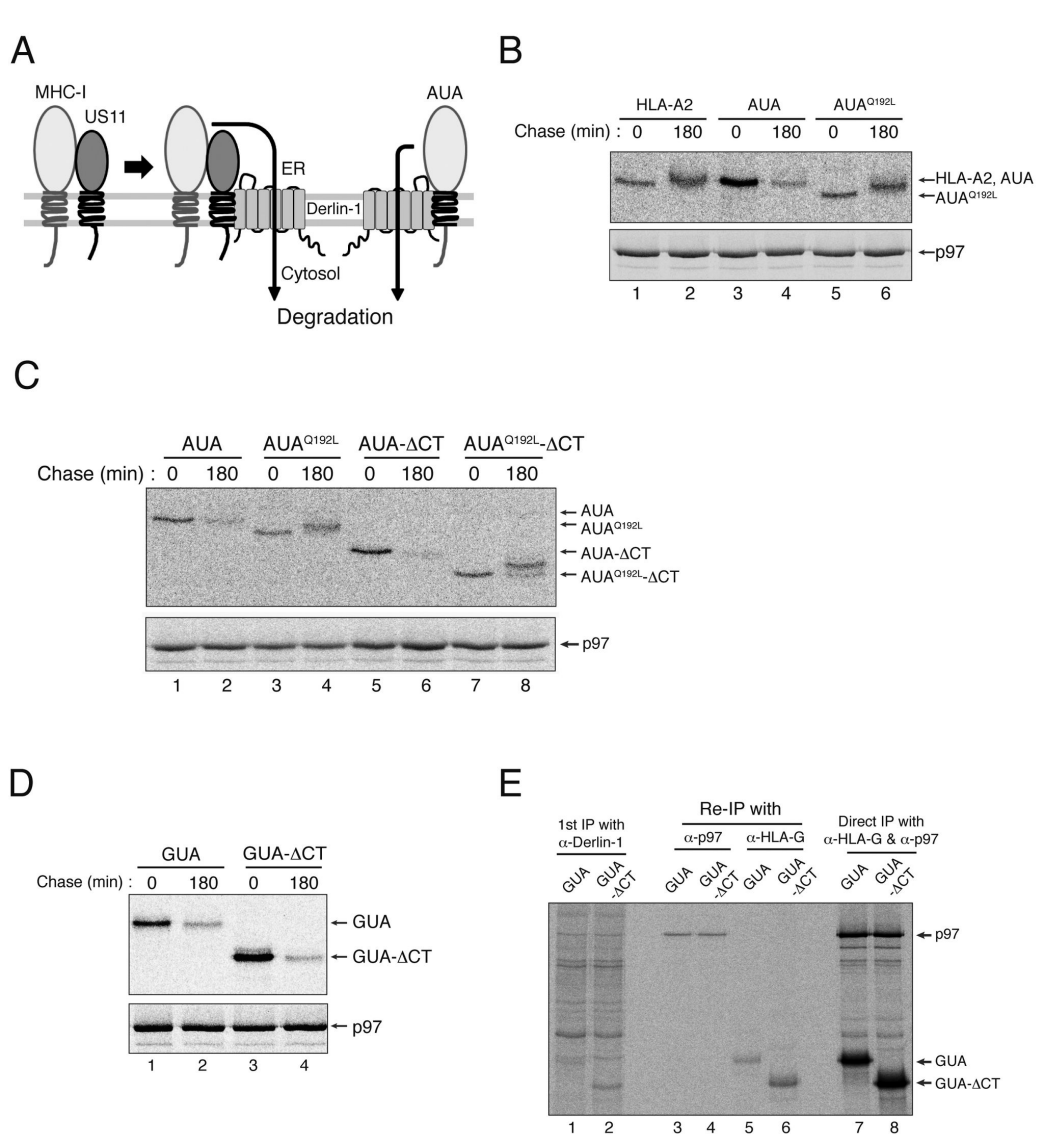
The MHC-I heavy chain cytosolic tail is not required for the degradation of MHC-I hybrids forced to interact with Derlin-1. (A) A schematic representation of the degradation of an MHC-I/US11 hybrid forced to interact with Derlin-1. (B) AUA is degraded after binding to Derlin-1. HeLa cells were transfected with wild-type HLA-A2, AUA or AUA^Q192L^, metabolically labeled for 15 min, and chased for 0 or 180 min. HLA-A2, AUA or AUA^Q192L^ was recovered by immunoprecipitation with mAb BB7.2, separated in SDS-PAGE gels, and analyzed by autoradiography. Immunoprecipitation of p97 (lower panel) indicates that the same amount of cell lysate was loaded into each lane of the gel. (C) The cytosolic tail of AUA is not required for its degradation. HeLa cells were transfected with AUA, AUA^Q192L^, AUA-ΔCT, or AUA^Q192L^-ΔCT, metabolically labeled for 15 min, and chased for 0 or 180 min. (D) Deletion of the MHC-I cytosolic tail does not block the degradation of GUA. HeLa cells were transfected with GUA or GUA-ΔCT, metabolically labeled for 15 min, and chased for 0 or 180 min. (E) Deleting the cytosolic tail from GUA does not abrogate its binding to Derlin-1. HeLa cells were transfected with GUA or GUA-ΔCT, metabolically labeled for 1 hr, lysed in 1% digitonin, and subjected to immunoprecipitation with an anti-Derlin-1 antibody. The precipitate was then boiled in SDS/DTT-containing buffer, diluted 10-fold in 1% NP-40, and then subjected to a second round of immunoprecipitation with the anti-p97 antibody or with mAb 4H84. All experiments were performed multiple times with similar results, and the data shown are representative of all results.

We recently reported that if MHC-I molecules are forced to interact with Derlin-1 by replacing their TMD with that of US11 (as depicted in Figure 5A), the resulting hybrid MHC-I molecules are exported to the cytosol and degraded by the proteasome in the absence of US11 [16]. As shown in Figure 5B, AUA (an HLA-A2/US11 chimera comprising the HLA-A2 ER luminal domain, the US11 TMD, and the cytosolic tail of HLA-A2) was largely degraded after 180 min; however, wild-type HLA-A2 remained stable (Figure 5B; compare lanes 1–2 and 3–4). This degradation is clearly dependent upon the interaction with Derlin-1, as AUA^Q192L^ (a chimera harboring a mutation that disrupts its interaction with Derlin-1) was completely resistant to degradation [16] (Figure 5B; lanes 5 and 6). If the cytosolic tail of the MHC-I heavy chain is required for US11-induced degradation because it is critical for strong interaction with Derlin-1, then deleting it may not affect the rate of AUA degradation because it has already associated with Derlin-1 via its US11 TMD. To test this hypothesis, we transfected HeLa cells with AUA or AUA-ΔCT and compared their half-lives in pulse-chase experiments (Figure 5C). The results showed that the degradation rate of AUA-ΔCT was comparable with that of AUA (Figure 5C; compare lanes 1–2 and 5–6), indicating that the degradation of AUA was not dependent upon the presence of its cytosolic tail. The same result was obtained for GUA, which comprises the HLA-G luminal domain, the US11 TMD, and the cytosolic tail of HLA-A2. Deleting the cytosolic tail did not delay the degradation of GUA (Figure 5D; compare lanes 1–2 and 3–4). The finding that the degradation of GUA is independent of the cytosolic tail can be well explained by the observation that deleting the cytosolic tail from GUA did not abrogate its interaction with Derlin-1 (Figure 5E; compare lanes 5 and 6). Consistent with this, deleting the C-terminal valine from AUA or GUA did not delay their degradation (data not shown). Taken together, these data strongly support the hypothesis that the cytosolic tail (or the C-terminal amino acid) of the MHC-I heavy chain is required for US11-induced degradation because it mediates the tight association between MHC-I molecules and Derlin-1 in the presence of US11.

### The C-terminal cytosolic region of Derlin-1 binds to MHC-I molecules

**Figure 6:**
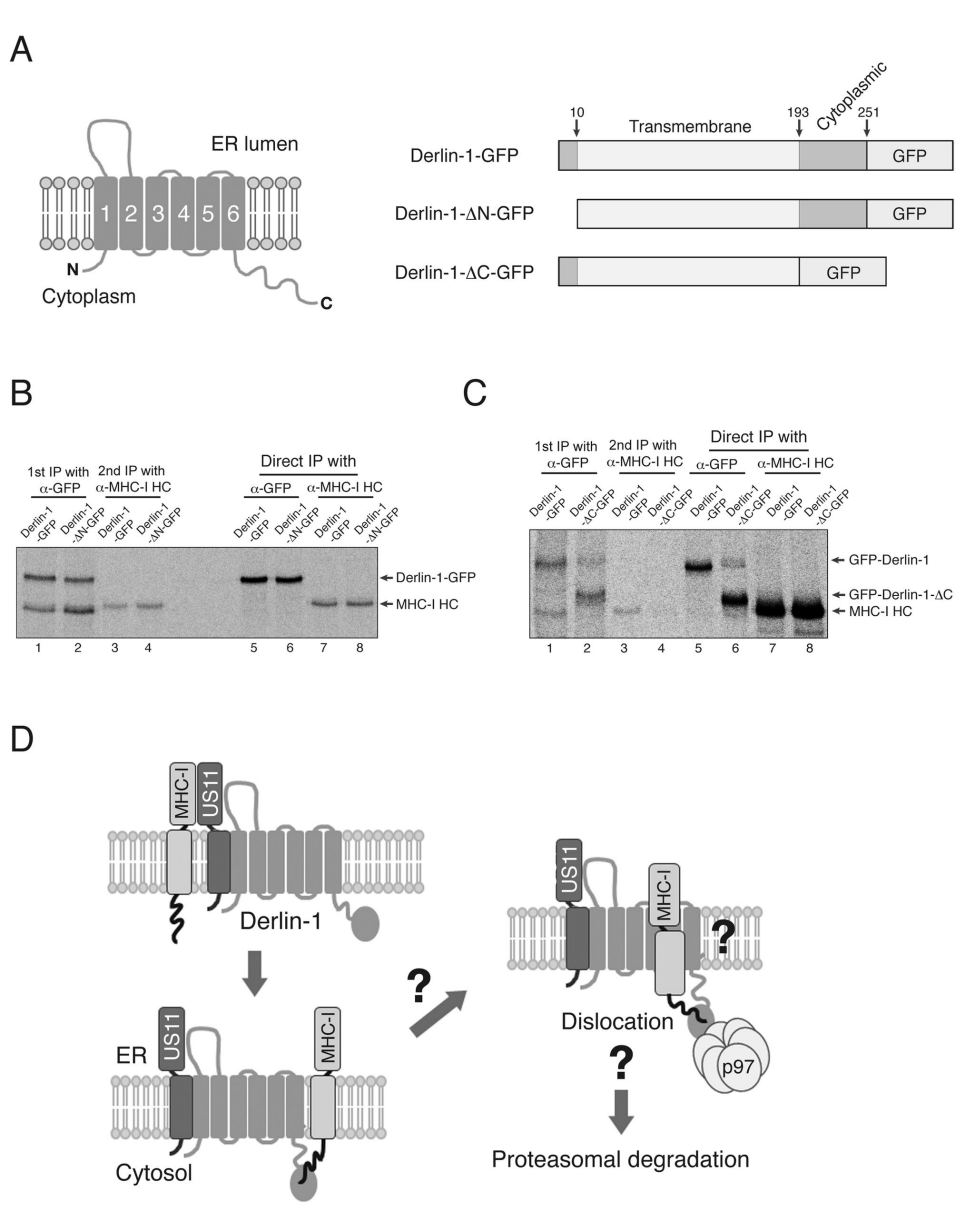
The C-terminal cytosolic region of Derlin-1 binds MHC-I molecules during US11-induced ERAD. (A) Proposed membrane topology of Derlin-1 (left) and a schematic representation of the GFP-conjugated Derlin-1 constructs (B) The N-terminal cytosolic region of Derlin-1 is not required for binding to MHC-I molecules. U373MG-US11 cells were transfected with Derlin-1-GFP or Derlin-1-ΔN-GFP, metabolically labeled for 1 hr, lysed in 1% digitonin, and subjected to immunoprecipitation with anti-GFP antibody. The precipitate was then boiled in SDS/DTT-containing buffer, diluted 10-fold in 1% NP-40, and subjected to a second round of immunoprecipitation with mAb HC10, which reacts with the denatured form of MHC-I heavy chains. (C) Deletion of Derlin-1 C-terminal region abolishes its interaction with MHC-I molecules. U373MG-US11 cells were transfected with Derlin-1-GFP or Derlin-1-ΔC-GFP, metabolically labeled for 1 hr, lysed in 1% digitonin, and subjected to immunoprecipitation with anti-GFP antibody. The precipitate was then boiled in SDS/DTT-containing buffer, diluted 10-fold in 1% NP-40, and subjected to a second round of immunoprecipitation with mAb HC10. All experiments were performed multiple times with similar results, and the data shown are representative of all results. (D) Working model describing the cytosolic interaction between Derlin-1 and MHC-I molecules during US11-induced ERAD. See the *DISCUSSION* section for a detailed explanation.

The above findings indicate that the cytosolic portion of Derlin-1 may be involved in its interaction with the MHC-I heavy chain during US11-induced degradation. According to the proposed membrane topology of Derlin-1 [15] (also see Figure 6A), both the N-terminal nine amino acids and the C-terminal region comprising 59 amino acids are exposed to the cytosol; thus, they are best placed to interact with the cytosolic tail of an MHC-I molecule. The two cytosolic loops connecting the second and third or the fourth and fifth TMDs seem to be too short to provide a large enough contact area for an MHC-I molecule. Therefore, to examine whether the N-terminal or C-terminal region of Derlin-1 interacted with MHC-I molecules, we constructed two deletion mutants of Derlin-1 (Figure 6A). In one mutant (Derlin-1-ΔN-GFP), eight of the nine amino acids that comprise the N-terminal cytosolic region were deleted. In the other (Derlin-1-ΔC-GFP), the entire C-terminal region was deleted. Deleting the N-terminal cytosolic region did not affect the interaction with MHC-I heavy chains (Figure 6B; compare lanes 3 and 4); however, deleting the C-terminal domain resulted in the near-complete loss of binding to MHC-I molecules (Figure 6C; compare lanes 3 and 4), This result suggests that the C-terminal region of Derlin-1 is responsible for binding to the cytosolic tail of MHC-I molecules during US11-induced degradation.

## Discussion

Based on our finding that the cytosolic tail of the MHC-I heavy chain binds to Derlin-1 and that this interaction determines MHC-I heavy chain degradation during US11-induced ERAD, we propose a working model that explains how HCMV US11 dislocates MHC-I molecules to the cytosol for proteasomal degradation (Figure 6D). In HCMV-infected cells, US11 captures MHC-I molecules and brings them to Derlin-1 via its direct binding to Derlin-1. The observation that the amount of US11 bound to Derlin-1 was comparable in GGA- and GGA-ΔCT-expressing cells (Figure 2D; compare lanes 1 and 2), but the amount of GGA-ΔCT bound to Derlin-1 was significantly less (Figure 2D; compare lanes 5 and 6), suggests that after delivering the MHC-I molecules to Derlin-1, US11 dissociates from the MHC-I molecule but remains bound to Derlin-1. During this transition, the C-terminal region of Derlin-1 may recognize and bind tightly to the cytosolic tail (or the C-terminal amino acid) of the MHC-I heavy chain. The MHC-I molecules bound to Derlin-1 are then dislocated to the cytosol via an as-yet unknown mechanism. Furthermore, the C-terminal region of Derlin-1 is also thought to interact with p97; thus, the Derlin-1-bound MHC-I molecules may also interact with p97, which then extract them from Derlin-1 and release them into the cytosol for degradation by the proteasome.

Is this cytosolic interaction between Derlin-1 and the substrate MHC-I molecule common to other ERAD pathways? A recent study shows that RHBDL4, an ER-resident rhomboid protease, interacts with membrane-bound protein substrates via its cytosolic ubiquitin binding domain [18]. Although Derlin-1, an inactive rhomboid protease, lacks a ubiquitin binding domain *per se*, it can associate with the Ufd1-Npl4 complex, which has ubiquitin-binding activity [32], via interaction with p97 [25]. Thus, the C-terminal cytosolic region of Derlin-1 may serve as a platform upon which ERAD substrates first encounter cytosolic ERAD factors. If this is the case, then the cytosolic interaction between Derlin-1 and its transmembrane protein substrates may be a common mechanism in ERAD pathways employing a subset of the rhomboid protease family proteins.

It is suggested that the cytosolic ATPase p97 interacts with MHC-I molecules by associating with Derlin-1 during US11-induced degradation [25,33] (also see Figures S2 and S3). If this is the case, then deleting the cytosolic tail should reduce the binding between MHC-I molecules and p97. However, we repeatedly observed that deleting the cytosolic tail (or the C-terminal amino acid) of the MHC-I heavy chain only causes a marginal reduction in its binding to p97 (Figure 2C; compare lanes 5 and 6). This may be because the interaction between the MHC-I heavy chain and p97 is extremely transient; thus, few p97/MHC-I complexes exist in the steady state. Furthermore, since p97-bound MHC-I molecules are likely to be degraded very quickly by the proteasome they cannot be easily immunoprecipitated. Consistent with this, we repeatedly observed that the amount of MHC-I heavy chains co-precipitated with p97 was much less than that of co-precipitated with Derlin-1 (Figure S2; compare lanes 5 and 11).

The present study showed that the C-terminal amino acid (valine or alanine) of the human MHC-I heavy chains is critical for US11-induced degradation. This amino acid appears to mediate the binding of MHC-I molecules to Derlin-1 only in the presence of US11; no detectable interaction was seen between Derlin-1 and MHC-I molecules in the absence of US11 [10]. This raises the question of whether the amino acid must be either valine or alanine, or whether it can be an amino acid belonging to the same group, such as a hydrophobic amino acid. The latter seems likely, as replacing the C-terminal valine of HLA-A2 with a hydrophilic amino acid, but not with another hydrophobic amino acid, such as isoleucine or phenylalanine, blocked HLA-A2 degradation (data not shown). Thus, the hydrophobic interaction between the C-terminal amino acid of the MHC-I heavy chain and the C-terminal region of Derlin-1 seems to mediate the interaction between these two proteins. We recently reported that the C-terminal amino acid of the MHC-I heavy chain functions as an ER export signal [27]; therefore, an interesting possibility is that MHC-I molecules must leave the ER before undergoing US11-induced degradation. Indeed, some ERAD pathways require proteins that are involved in ER export [34–36]. However, ER export is unlikely to be necessary for US11-induced ERAD because brefeldin A, which impedes the early secretory pathway, failed to block US11-induced degradation [21]. Moreover, overexpression of the GDP-locked Sar1-T39N mutant or Syntaxin-5 did not inhibit US11-induced degradation (data not shown). Sar1 is an essential component of the COPII complex, which is required for ER-to-Golgi trafficking; thus, overexpression of the Sar1-T39N mutant, which functions in a dominant-negative manner, prevented anterograde COPII vesicle-mediated ER export [37]. Synatixin-5 is a SNARE protein, which is required for the fusion of COPII vesicles with the Golgi membrane; thus, overexpression of Syntaxin-5 inhibited ER-to-Golgi trafficking by causing an imbalance in the stoichiometry of functional SNARE proteins [38].

Many viruses have evolved strategies to evade immune surveillance by preventing MHC-I-mediated antigen presentation. Because MHC-I molecules are highly polymorphic, and viruses produce only a limited number of immune-modulating proteins, viruses are likely to target common/conserved regions within the different MHC-I alleles or proteins that are required for MHC-I-mediated antigen presentation, such as TAP and tapasin [39–41]. The results of the present study suggest that HCMV US11 may take advantage of the interaction between Derlin-1 and the C-terminal amino acid of the MHC-I heavy chain. Interestingly, the C-terminal region of Derlin-1 does not seem to have an intrinsic ability to interact with the cytosolic tails of MHC-I heavy chains; no interaction was observed between Derlin-1 and the MHC-I heavy chain in the absence of US11 [10]. Furthermore, in vitro binding studies showed that the cytosolic tail of HLA-A2 showed no affinity for full-length Derlin-1 or C-terminal cytosolic region of Derlin-1 expressed and purified from *E. coli* (data not shown). Thus, these results indicate that US11 may modulate the substrate specificity of Derlin-1 such that Derlin-1 recognizes and binds to the cytosolic tail (or the C-terminal amino acid) of the MHC-I heavy chain. Indeed, US11 remains bound to Derlin-1 even after the MHC-I molecules dissociate from US11 (Figure 2D and Figure 3D). This is further supported by the observation that, in the absence of US11, the MHC-I cytosolic tail was not necessary for degradation of AUA or GUA, which was forced to bind to Derlin-1 via the US11 TMD. HIV Nef may use a similar mechanism to drive MHC-I molecules from the trans-Golgi network to the lysosome for degradation [42]. Nef appears to modulate the binding specificity of clathrin adaptor protein complex 1 (AP1), which does not normally recognize MHC-I molecules, such that AP1 binds to the conserved tyrosine residue within the cytosolic tails of MHC-I molecules and recruits them into clathrin-coated vesicles for delivery to the lysosome.

Despite the central role played by Derlin-1 during ERAD, little is known about how Derlin-1 interacts with ERAD substrates prior to or during their dislocation to the cytosol. The present study used US11-induced degradation of MHC-I molecules as a model system to examine Derlin-1-depedent ERAD pathways. The results show that Derlin-1 recruits MHC-I molecules from US11 via the binding of its C-terminal cytosolic region to the cytosolic tail of the MHC-I heavy chain. Furthermore, this interaction is critical for the dislocation of the MHC-I heavy chains to the cytosol for proteasomal degradation. To the best of our knowledge, this is the first study to show that Derlin-1 recognizes and captures its transmembrane protein substrates via its C-terminal cytosolic region; thus, the current findings may provide novel functional insights into how the rhomboid protease family proteins interact with their ERAD substrates.

## Supporting Information

Figure S1 **Deletion of a single C-terminal amino acid renders MHC-I heavy chains resistant to US11-induced degradation.**
(A) Deletion of the cytosolic tail or the C-terminal valine renders HLA-A2 resistant to US11-induced degradation. U373MG-US11 cells were transfected with wild-type HLA-A2, HLA-A2-ΔV or HLA-A2-ΔCT, metabolically labeled for 15 min, and then chased for 0 or 60 min. HLA-A2, HLA-A2-ΔCT, or HLA-A2-ΔV was recovered by immunoprecipitation with mAb HCA2, separated by SDS-PAGE, and analyzed by autoradiography with a phosphorimager. Immunoprecipitation of HSP90 (lower panel) shows that the same amount of cell lysate was loaded into each lane of the gel. (B) Deleting the C-terminal amino acid from the GGA construct blocks its degradation by US11. U373MG-US11 cells were transfected with GGA or GGA-ΔV, metabolically labeled for 15 min, and then chased for 0 or 90 min. GGA or GGA-ΔV was then recovered by immunoprecipitation with mAb 4H84, separated by SDS-PAGE, and analyzed by autoradiography. Comparable levels of HSP90 show that the amount of the cell lysate used was equal between samples (lower panel).
(TIF)

Figure S2 **The amount of MHC-I heavy chains co-precipitated with p97 is significantly less than that of co-precipitated with Derlin-1 in US11-expressing cells.**
U373MG-US11 cells were metabolically labeled with ^35^S-methionine/cysteine for 1 hr, lysed in 1% digitonin, and then subjected to immunoprecipitation with anti-Derlin-1 antibody, anti-VIMP antibody, or anti-p97 antibody (lanes 1–3). The precipitates were then boiled in SDS/DTT-containing buffer to disrupt all protein–protein interactions, diluted 10-fold in 1% NP-40, and then subjected to a second round of immunoprecipitation with the anti-Derlin-1 antibody, the anti-VIMP antibody, the anti-p97 antibody, or mAb HC10. MHC-I heavy chains precipitated by mAb HC10 were further incubated at 37° C in the presence or absence of EndoH. The samples were then separated in 12% SDS-PAGE gels and analyzed by autoradiography.
(TIF)

Figure S3 **Interaction between p97 and MHC-I heavy chains is much stronger in US11-expressing cells than in control cells.**
U373MG control cells or 373MG-US11 cells were transfected with GGA, metabolically labeled with ^35^S-methionine/cysteine for 1 hr, lysed in 1% digitonin, and then subjected to immunoprecipitation with anti-p97 antibody (lanes 1 and 2). The precipitates were then boiled in SDS/DTT-containing buffer to disrupt all protein–protein interactions, diluted 10-fold in 1% NP-40, and then subjected to a second round of immunoprecipitation with the anti-p97 antibody (lanes 3 and 4) or mAb 4H84 (lanes 5 and 6). The samples were then separated in 10% SDS-PAGE gels and analyzed by autoradiography.
(TIF)
