# Supplementary figures and images for: The C-Terminal Amino Acid of the MHC-I Heavy Chain Is Critical for Binding to Derlin-1 in Human Cytomegalovirus US11-Induced MHC-I Degradation
Source: PLoS One. 2013 Aug 12;8(8):e72356. doi: 10.1371/journal.pone.0072356 (PMC3741148; doi:10.1371/journal.pone.0072356)

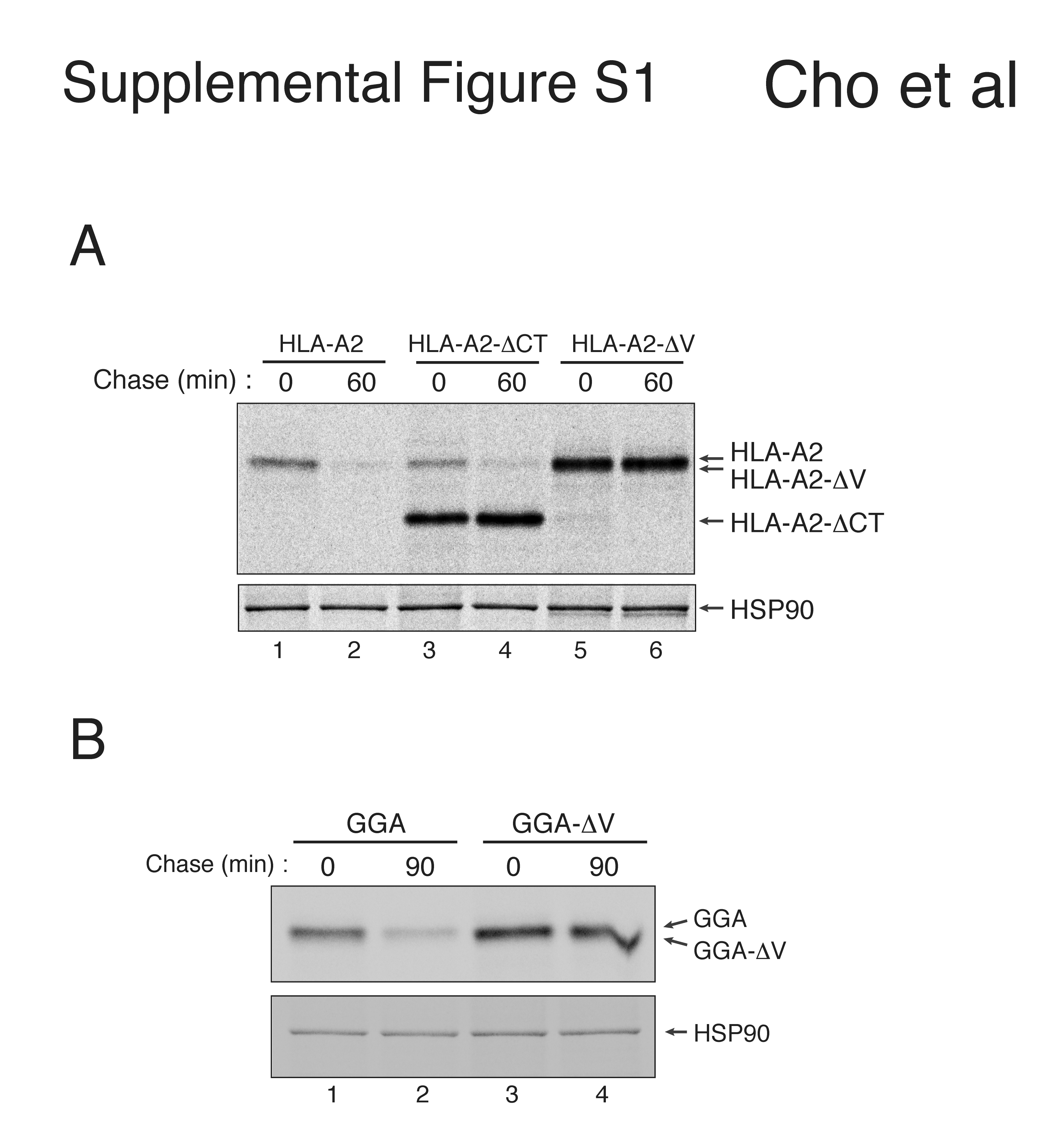

Supplement: Figure S1 — Deletion of a single C-terminal amino acid renders MHC-I heavy chains resistant to US11-induced degradation. (A) Deletion of the cytosolic tail or the C-terminal valine renders HLA-A2 resistant to US11-induced degradation. U373MG-US11 cells were transfected with wild-type HLA-A2, HLA-A2-ΔV or HLA-A2-ΔCT, metabolically labeled for 15 min, and then chased for 0 or 60 min. HLA-A2, HLA-A2-ΔCT, or HLA-A2-ΔV was recovered by immunoprecipitation with mAb HCA2, separated by SDS-PAGE, and analyzed by autoradiography with a phosphorimager. Immunoprecipitation of HSP90 (lower panel) shows that the same amount of cell lysate was loaded into each lane of the gel. (B) Deleting the C-terminal amino acid from the GGA construct blocks its degradation by US11. U373MG-US11 cells were transfected with GGA or GGA-ΔV, metabolically labeled for 15 min, and then chased for 0 or 90 min. GGA or GGA-ΔV was then recovered by immunoprecipitation with mAb 4H84, separated by SDS-PAGE, and analyzed by autoradiography. Comparable levels of HSP90 show that the amount of the cell lysate used was equal between samples (lower panel). (TIF) [file pone.0072356.s001.tif]

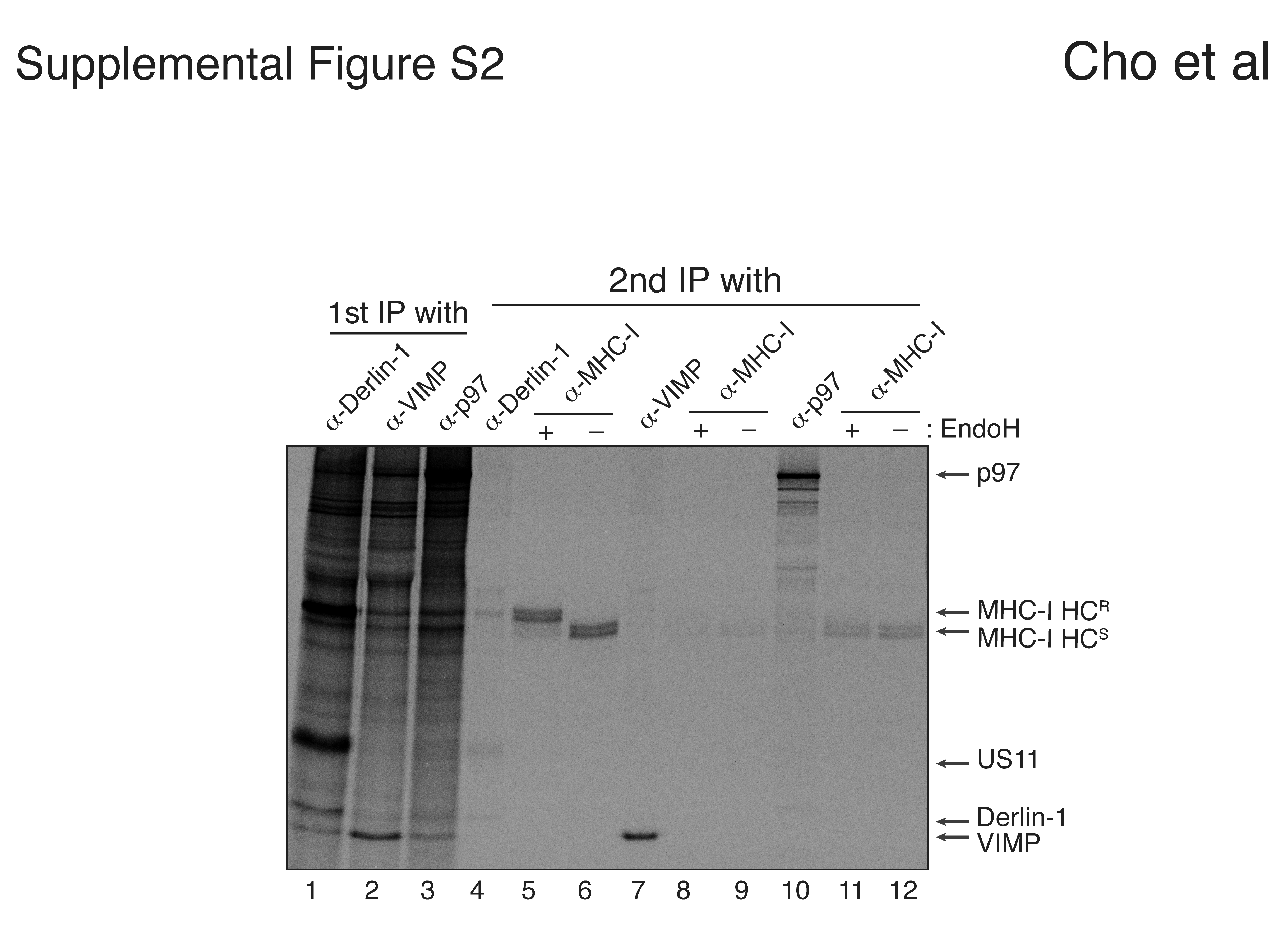

Supplement: Figure S2 — The amount of MHC-I heavy chains co-precipitated with p97 is significantly less than that of co-precipitated with Derlin-1 in US11-expressing cells. U373MG-US11 cells were metabolically labeled with 35S-methionine/cysteine for 1 hr, lysed in 1% digitonin, and then subjected to immunoprecipitation with anti-Derlin-1 antibody, anti-VIMP antibody, or anti-p97 antibody (lanes 1–3). The precipitates were then boiled in SDS/DTT-containing buffer to disrupt all protein–protein interactions, diluted 10-fold in 1% NP-40, and then subjected to a second round of immunoprecipitation with the anti-Derlin-1 antibody, the anti-VIMP antibody, the anti-p97 antibody, or mAb HC10. MHC-I heavy chains precipitated by mAb HC10 were further incubated at 37° C in the presence or absence of EndoH. The samples were then separated in 12% SDS-PAGE gels and analyzed by autoradiography. (TIF) [file pone.0072356.s002.tif]

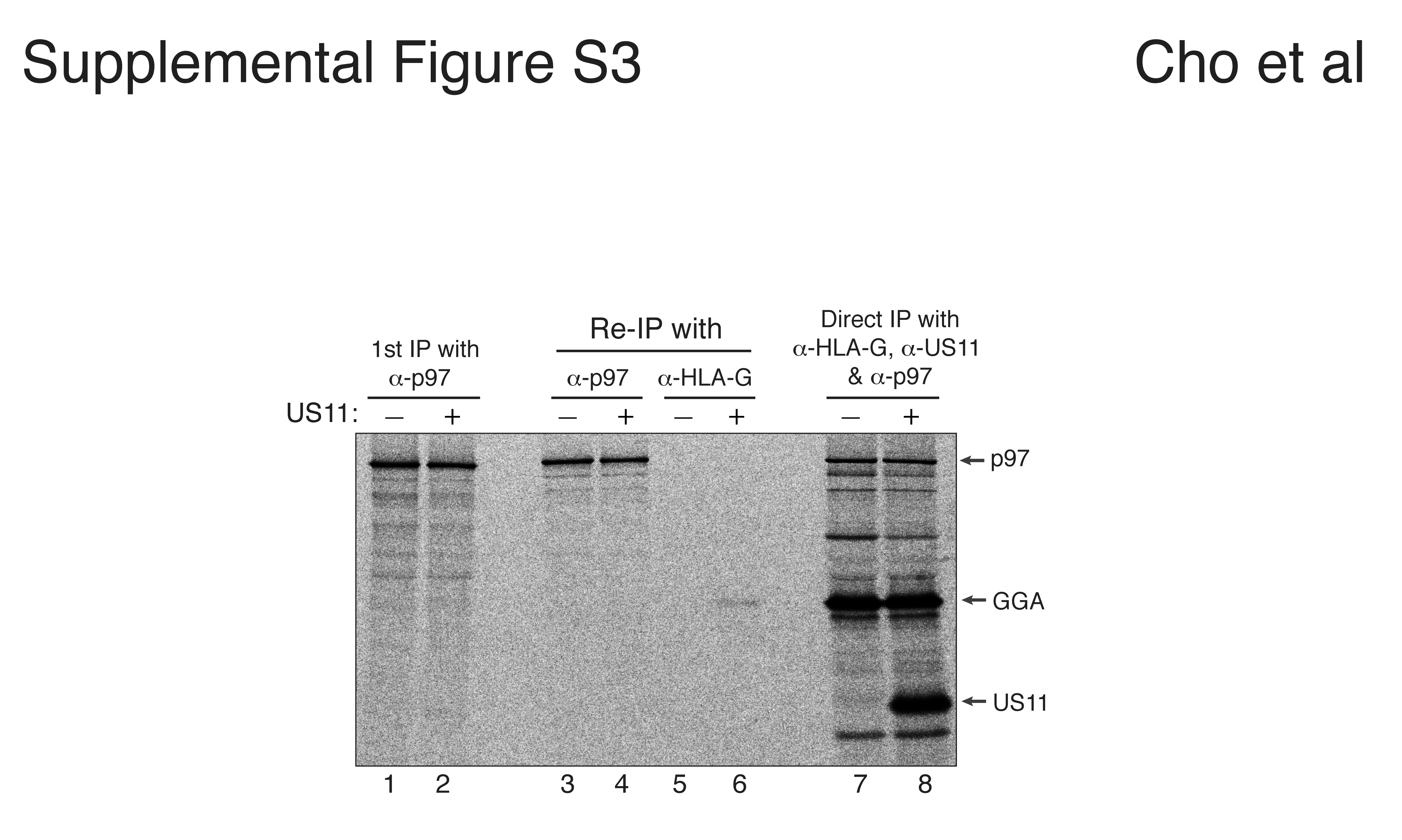

Supplement: Figure S3 — Interaction between p97 and MHC-I heavy chains is much stronger in US11-expressing cells than in control cells. U373MG control cells or 373MG-US11 cells were transfected with GGA, metabolically labeled with 35S-methionine/cysteine for 1 hr, lysed in 1% digitonin, and then subjected to immunoprecipitation with anti-p97 antibody (lanes 1 and 2). The precipitates were then boiled in SDS/DTT-containing buffer to disrupt all protein–protein interactions, diluted 10-fold in 1% NP-40, and then subjected to a second round of immunoprecipitation with the anti-p97 antibody (lanes 3 and 4) or mAb 4H84 (lanes 5 and 6). The samples were then separated in 10% SDS-PAGE gels and analyzed by autoradiography. (TIF) [file pone.0072356.s003.tif]
